# SUMOgo: Prediction of sumoylation sites on lysines by motif screening models and the effects of various post-translational modifications

**DOI:** 10.1038/s41598-018-33951-5

**Published:** 2018-10-19

**Authors:** Chi-Chang Chang, Chi-Hua Tung, Chi-Wei Chen, Chin-Hau Tu, Yen-Wei Chu

**Affiliations:** 1 School of Medical Informatics, Chung-Shan Medical University, Taichung, Taiwan; 2 IT Office, Chung Shan Medical University Hospital, Taichung, Taiwan; 3 Department of Bioinformatics, Chung-Hua University, Rm. S116, 707, Sec. 2, WuFu Rd., Hsinchu, 30012 Taiwan; 4 Department of Computer Science and Engineering, National Chung-Hsing University, 250, Kuo Kuang Rd., Taichung, 402 Taiwan; 5 Institute of Genomics and Bioinformatics, National Chung Hsing University, 250, Kuo Kuang Rd., Taichung, 402 Taiwan; 6 Biotechnology Center, Agricultural Biotechnology Center, Institute of Molecular Biology, National Chung Hsing University, 250, Kuo Kuang Rd., Taichung, 402 Taiwan

## Abstract

Most modern tools used to predict sites of small ubiquitin-like modifier (SUMO) binding (referred to as SUMOylation) use algorithms, chemical features of the protein, and consensus motifs. However, these tools rarely consider the influence of post-translational modification (PTM) information for other sites within the same protein on the accuracy of prediction results. This study applied the Random Forest machine learning method, as well as motif screening models and a feature selection combination mechanism, to develop a SUMOylation prediction system, referred to as SUMOgo. With regard to prediction method, PTM sites were coded as new functional features in addition to structural features, such as sequence-based binary coding, encoded chemical features of proteins, and encoded secondary structure information that is important for PTM. Twenty cycles of prediction were conducted with a 1:1 combination of positive test data and random negative data. Matthew’s correlation coefficient of SUMOgo reached 0.511, which is higher than that of current commonly used tools. This study further verified the important role of PTM in SUMOgo and includes a case study on CREB binding protein (CREBBP). The website for the final tool is http://predictor.nchu.edu.tw/SUMOgo.

## Introduction

Post-translational modification (PTM) of proteins refers to the chemical modification of proteins after their translation^1–3^. After PTM, amino acids may attach to another biochemical functional group of proteins (e.g. acetates, phosphates, lipids, carbohydrates, etc.), and their chemical properties or structure may thus change, expanding the functions of these proteins.

Small ubiquitin-like modifier (SUMO) is a type of protein that results in the SUMOylation of amino acids. SUMOylation is a reversible PTM that differs from ubiquitin in its functions and surface charge despite a 20% structural similarity with ubiquitin^4^. SUMO can be found in eukaryotes in yeast, plants, and vertebrates and has an important role in biomechanical processes, such as gene expression, DNA repair, chromosome recombination, and cell signaling^4–6^. SUMOylation regulation and control is related to many kinds of diseases, for example, neurodegenerative diseases^7^, congenital heart defects^8^, diabetes^9^, and cancer^10^. Therefore, the identification of potential SUMOylation sites has benefits for research on various diseases and biomechanisms.

An earlier study on SUMOylation indicated that SUMO binds after the consensus motif ψ-K-x-E, where ψ is a hydrophobic amino acid (I, V, L, A, P, or M), K is lysine, x is an arbitrary amino acid, and E is glutamic acid. This consensus motif was observed as the most common in the earlier study, with only 23% (56/239) of SUMO sites not matching the consensus motif^11^. Another study also indicated that ψ-K-x-E\D (where D is an aspartic acid)^12,13^ was the consensus motif and reported that 26% (69/268) of SUMO sites did not follow this consensus motif^14^. Most studies on SUMOylation have used these two values as reference points. A more recent study in 2014, however, indicated that ~40% (400/983) of SUMOylation sites do not follow this consensus motif but instead have the consensus motif (A, F, G, I, L, M, P, V, Y)Kx(E/D)^1^, which suggests that we may be lacking critical information about SUMOylation.

Lysine is one of 20 common amino acids. Because of its physical and chemical properties, it can interact with several proteins or substrates. With respect to PTM, lysine is not only the most frequently modified amino acid but also the one subjected to the widest range of PTMs, which include SUMOylation. Moreover, lysine accounts for the majority of SUMOylation sites, with few exceptions^6^.

Previously, biologists needed to conduct complicated experiments with the use of expensive materials to determine the PTM of a protein. The development of bioinformatics in recent years has allowed researchers to make predictions about the PTM of a protein by integrating informatics, mathematics, and statistics. Because of the reversibility of SUMOylation, repeated experiments are required in the absence of SUMOylation site modification, which increases the importance of predictive screens for potential sites.

Most current prediction tools involve the analysis of chemical properties and consensus motifs of protein sequences or the use of specific algorithms to predict SUMOylation sites. For example, the PCI-Based Sumo Site Prediction Server (PCI-SUMO) is used to predict SUMOylation sites with the parallel cascade identification (PCI) algorithm^15^. The web servers SUMOsp^11^ and GPS-SUMO^1^ were developed using group-based prediction system (GPS) and its updated version, respectively, for the prediction of SUMOylation sites and SUMO-interaction motifs (SIMs); in addition, the recently developed tool JASSA^2^ is used to search for SUMOylation sites and SIMs based on the unique position frequency matrix scoring system. However, research on these tools used for predicting SUMOylation sites revealed that most of them failed to consider the PTM of other proteins. Only one study on prediction tools has indicated the potential effect of acetylation on SUMOylation and suggested its importance^16^. As a starting point, this raises the issue of the potential impacts of the PTM of other sites within the same protein on SUMOylation. A review of the literature revealed only one study that analyzed the competition between SUMOylation and acetylation at the same SUMOylation site and suggested the importance of the secondary structure of the protein in this process^17^. Thus, based on this competition resulting from acetylation at a SUMOylation site, this study also raised the question of whether other protein PTMs can compete with SUMOylation.

Machine learning is a common method currently used to resolve SUMOylation sites^16,18–20^. This study applied the Random Forest machine learning method, as well as motif screening models and a feature selection combination (FSC) mechanism, to develop a SUMOylation prediction system. This study also used a support vector machine (SVM) to filter the parameters and conditions of the prediction model. Our research finally developed a SUMOylation prediction tool, named SUMOgo, which we used to explore whether such competition can affect the accuracy of SUMOylation prediction tools and whether the rules of other PTMs can be applied to SUMOylation. In this study, WEKA data mining and machine learning software were used to determine the most optimal machine learning algorithm^21^.

In this study, we verified the predictive power of SUMOgo through independent testing set and compared the results from SUMOgo with those from other prediction tools such as GPS-SUMO^1^, SUMOsp2.0^11^, JASSA^2^, and PCI-SUMO^15^. Within the independent testing data set, there were 867 positive sets and 18825 negative sets collected to detect the accuracy of overall prediction. This study used Matthews correlation coefficient (MCC) to test the positive-negative correlation. The results showed that the prediction accuracy of SUMOgo is greater than that of other SUMOylation site prediction tools with an average MCC of up to 0.511. In addition, SUMOgo was applied to both conserved-motif and nonconserved-motif screening models to reach separate predictions. Trained to predict their specific data, the prediction results of motif screening models showed good performance. This research also demonstrated that excluding post-modification distributions in the feature selection can affect the accuracy of the predictions. We also provide a practical case study to show how SUMOgo can be used to predict the potential SUMOylation sites of CREB binding protein (CREBBP). Our research has resulted in a user-friendly web server that is freely available for researchers at http://predictor.nchu.edu.tw/SUMOgo.

## Methods

### Preparation of the data set

The experimental data used in this study were derived from three protein databases, UniProt^22^, dbPTM^23^, and PhosphoSitePlus^24^. The SUMOylation data from UniProt were used as the training data set, whereas data from dbPTM and PhosphoSitePlus (excluding the data from UniProt) were used as the testing data set. Once the training and testing data sets were defined, the training data set was run through CD-HIT to remove sequences showing high similarity. The CD-HIT sequence identity cut-off values in this study were set to 0.3 and 0.6^25–27^. The target in this study included all lysines in amino-acid segments. Based on the data regarding SUMOylation sites, SUMOylation sites with lysine as the amino acid were set as positive and non-SUMOylation sites with lysine were set as negative. Meanwhile, negative sites were selected from proteins which at least one positive site existed. Under the CD-HIT sequence identity cut-off value of 0.3, the number of lysines that are part of a SUMOylation site in the training data set was 869. At the cut-off value of 0.6, the total positive number of lysines in the training data set was 1166. On the other hand, the quantities of negative data in the training data set were 20903 (with cut-off 0.3) and 26169 (with cut-off 0.6) respectively. In the testing data set, there were 867 positive data and 18825 negative data respectively.

As the approximate positive-negative data ratio in the testing data set was 1:20, that is, the proportion of negative data was higher than that of positive data, the prediction tools were likely to be more accurate in predicting negative data, which could affect the MCC results. To avoid this phenomenon, this study compiled a new testing data set by conducting 20 cycles of random extraction of negative data at a 1:1 ratio from the testing data set. The final results were presented in terms of the mean MCC. To prevent the higher accuracy of a single positive or negative data set from affecting reliability because of the overstated accuracy of overall prediction, MCC was used as an objective index of accuracy.

### Consensus motif classification

The consensus motif ψ-K-x-E is used in most modern prediction tools. However, some studies consider hydrophobic amino acids L, I, V, and F^18^ or I, V, L, A, P, M, G, and Y for ψ. In addition to the difference in hydrophobic amino acids, there exists a different consensus motif, ψ-K-x-E/D^20^. Therefore, six forms coded as C1–C6 were tested in this study, as shown in Table 1. In total, two models were built: one model followed the consensus motif, whereas the other model did not. The model matching the consensus motif was coded as CY. The model not matching the consensus motif was coded as CN.

**Table 1:** Consensus motif types.

Motif type	Motif form
C1	(L, V, M, F)Kx(E, D)
C2	(A, I, P, L, V, M, F)Kx(E, D)
C3	(A, I, P, L, V, M, F, G, Y)Kx(E, D)
C4	(L, V, M, F)Kx(E)
C5	(A, I, P, L, V, M, F)Kx(E)
C6	(A, I, P, L, V, M, F, G, Y)Kx(E)

Positive and negative data sets within the training and testing data sets were divided based on classification models. The extracted categories (C1-C6) included positive data sets matching the consensus motif, named as CY_P, and negative data sets matching the consensus motif, named as CY_N. In addition, positive and negative data sets not matching the consensus motif were named CN_P and CN_N, respectively. We refer to this procedure for motif screening models as the CNCY system.

### Architecture of SUMOgo

**Figure 1:**
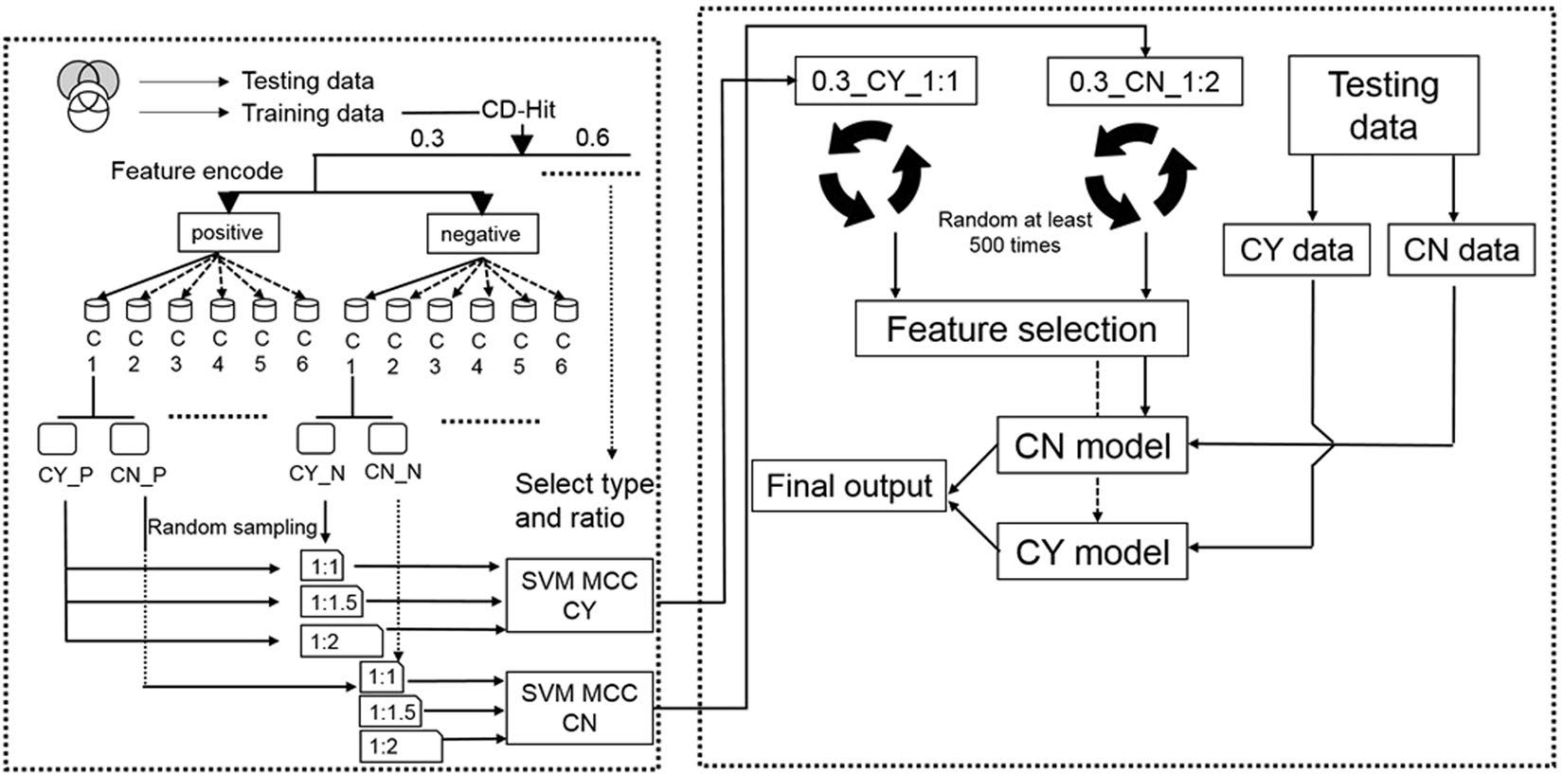
The experimental architecture of SUMOgo.

Figure 1 shows the main research procedures in this study, with the starting point at the upper left corner. The data from three databases (see “Preparation of the data set” section) was processed, deduplicated, and divided into the training data set and testing data set. The training data set was run through CD-Hit to remove similar sequences and was divided based on the cut-off values of 0.3 and 0.6. Each cut-off value separated data into positive and negative data sets, which were further divided into positive and negative data sets that follow (CY) or do not follow (CN) the consensus motif with six forms coded as C1–C6 (CY_P, CN_P, CY_N, and CN_N, where P indicates positive and N indicates negative).

A comparison of consensus motif types and the positive and negative data set ratio (P/N ratio) is necessary prior to the construction of the prediction model. Positive and negative data sets with different types of consensus motifs were constituted for the SVM learning. The entire positive data set was included in the SVM files, whereas a subset of the negative data was selected randomly to prevent the larger size of the negative data set from causing overlearning related to negative data in the SVM. The P/N ratios in this study were 1:1, 1:1.5, and 1:2. Each ratio of each consensus motif type was evaluated by 30 rounds of five-fold cross-validation. The results were presented in terms of mean MCC values. Positive and negative data sets with different proportions of CN and CY were combined into SVM learning for the calculation of the average MCC for each item after prediction and for constructing motif screening models.

Motif screening models were constructed based on the selected consensus motif type and ratio. The type selected finally in this study was “(L, V, M, F)Kx(E, D)”, referred to as motif type C1, and the P/N ratio was 1:1 for the CY model matching the consensus motif and 1:2 for the CN model not matching the consensus motif (see Results). During model construction, the ratio number was randomly selected from the entire negative data set. After selecting the optimal settings from the model set, ≥500 randomly selected and organized SVM models were established, among which the optimal model in the system was determined.

Feature selection was then conducted to determine whether the model can optimized. Feature selection and optimization was followed by a comparison of SVM prediction results and WEKA algorithm results to select the optimal algorithm. Finally, the testing data set was divided into data that follow (CY) and do not follow (CN) consensus rules to predict corresponding models.

To justify the CNCY system, this study compared the prediction results of motif screening models and investigated whether the separate application of CN and CY motif screening models would increase the overall MCC. Furthermore, to examine the effect of other PTMs on prediction accuracy and determine their importance for the prediction system, this study compared the accuracy of prediction models that included and did not include PTM features.

### Window size definition

For positive and negative data used in this study, lysine sites were selected from the protein sequences, and 10 amino acids upstream and downstream from the lysine site were extracted. Missing amino acids were substituted with “−“. A region of 21 amino acids with a lysine at the center position was thus set as the window size in this study. Lysine was set at the center because of frequent contacts between SUMOylation and ubiquitination and the lysine side chain of proteins. Moreover, one study reported a relationship between PTMs and lysine^28^. The window size of 21 was determined based on another study that proposed that the motif distance between acetylation and SUMOylation could not exceed 21 amino acids^17^.

### Feature coding

The methods used in this study included binary coding^11,29^ and the prediction of secondary structure using NetsurfP; in addition, six PTM sites were obtained using ModPred and 10 protein features were suggested for research. Among the 10 recommended values, five were obtained from AAindex (amino acid index database)^30,31^ and five were obtained from SWISSPROT and dbGET^32^. Table 2 shows the distribution and vector numbers of these features.

**Table 2:** Feature distribution and vector numbers.

Feature	Binary	AAindex + SWISSPROT/DbGET	NetsurfP	ModPred
Total bits	21 × 20 = 420	21 × 10 = 210	21 × 7 = 147	21 × 6 = 126
Position	1–420	421–630	631–777	778–903

#### Sequence-based features

Machine learning normally presents data in terms of vectors. Therefore, 20 dimensional vectors were used for the coding of 20 amino acids and the Gap. The dimension of these amino acids was set to 1. Thus, binary coding was used to convert 20 amino acids into 20 different numerical sequences of 0 and 1. Missing values (i.e., gaps) were substituted with 0. In total, 20 dimensional vectors multiplied by 21 were used. Amino acid sequence similarity was set as a feature in machine learning.

Most previous studies used AAindex^33^, an amino acid index and mutation matrix, to code physicochemical features. Over 500 defined physical and chemical properties of amino acids were recorded. Big data resulting from the excessive number of features can result in prolonged calculations and difficulties in improving and influencing machine learning outcomes. Therefore, this study referred to two studies and divided all features into ten large categories. For instance, William *et al*.^34^ simplified amino acid AAindex features based on their similarity and divided them into five categories, namely polarity, secondary structure, molecular size or volume, codon diversity, and electrostatic charge. Mathura *et al*.^35^ did statistical analysis from the literatures and distinguished the following categories of features: hydrophobicity, side chain length, α-helix propensity, number of codons, and β-strand propensity. The categories of amino acid features proposed in both of these studies were incorporated in the coding, and thus each amino acid was presented using 10 values. Twenty amino acid numbers corresponding to ten feature categories were used in coding. In total, 10 dimensional vectors multiplied by 21 were applied.

#### Structure-based features

Functional configurations in proteins are formed through the folding of amino acid peptides. The location of amino acid sites on the interior or exterior surface of proteins is particularly important in research on interaction effects and the tertiary structure of proteins. The catalysis of enzyme-substrate complexes and structure surface accessibility may also have an effect. This study used the NetSurfP prediction website to gather surface accessibility data for individual amino acids, which were divided into seven types: (1) buried or exposed amino acid; (2) relative surface accessibility; (3) absolute surface accessibility; (4) predicted Z-score of surface area; (5) α-helix probability score; (6) β-strand probability score; and (7) coil probability score. Seven dimensional vectors multiplied by 21 were used in this coding step.

#### Function-based features

ModPred is a tool that can simultaneously predict 23 types of protein PTMs. In this study, sites with a low confidence level were coded as 10, sites with a moderate confidence level were coded as 50, sites with a high confidence level were coded as 100, and sites without post-modification were coded as 0. Based on the research on lysine-related PTMs^28^, six protein PTMs were selected from this website for prediction, which included acetylation, hydroxylation, methylation, phosphorylation, SUMOylation, and ubiquitination. Six dimensional vectors multiplied by 21 were used in this coding step.

### Feature selection combination (FSC)

Past studies evaluated prediction models based only on their accuracy, which could lead to the phenomenon of overfitting and to better accuracy of the prediction model during its training test and inaccuracy with respect to the independent testing data prediction results that are later obtained. Therefore, this study integrated the LIBSVM^36^ and mRMR^37^ feature selection methods to form the FSC mechanism, to examine the importance of training data in the two models with regard to features. LIBSVM feature selection calculations provide all features with an F-score to indicate their importance. The ascending priority based on F-score values is used to test the accuracy of different feature numbers. The mRMR feature selection method is based on the principle of minimum redundancy and sorts features in ascending order based on their importance.

### Algorithm selection

Previous studies used the SVM algorithm to derive prediction model parameters. However, to test the applicability of other machine learning and data mining algorithms, this study used WEKA to evaluate six categories of algorithms, namely tree, rule, meta, lazy, function, and baye, and determine the most effective machine learning method. The different algorithms provided by WEKA were compared and prediction methods were evaluated using five-fold cross-validation.

### Evaluation measures

To assess the predictive performance of the classifier, we used the following formulas. TP, FP, FN, and TN represent a true positive, false positive, false negative, and true negative, respectively. Sensitivity (Sn), also called the true positive rate, reflects the percentage of correct predictions of SUMOylation. Specificity (Sp) or the true negative rate indicates the percentage of correct predictions of non-SUMOylation sites. Accuracy (ACC) is used to assess the overall predictive power of the prediction accuracy. MCC values range from –1 to 1, of which a value of 1 represents a completely correct prediction, a value of 0 represents a random prediction, and a value of –1 represents exactly the opposite prediction.

1 $$Sn=\frac{TP}{(TP+FN)}\times 100 \% $$

2 $$Sp=\frac{TN}{(TN+FP)}\times 100 \% $$

3 $$Precision=\frac{TP}{(TP+FP)}\times 100 \% $$

4 $$ACC=\frac{TP+TN}{TP+FP+TN+FN}\times 100 \% $$

5 $$MCC=\frac{(TP\times TN)-(FN\times FP)}{\sqrt{(TP+FN)\times (TN+FP)\times (TP+FP)\times (TN+FN)}}$$

## Results and Discussion

### Consensus motif type selection

**Figure 2:**
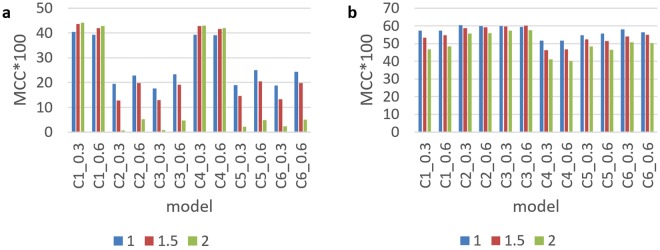
Performance of using different consensus motif types. (**a**)The average MCC for each of the CN models. (**b**) The average MCC for each of the CY models.

As shown in Fig. 2a, the C1 and C4 mean MCC values in the CN model were substantially higher than those for other models. Mean MCC values at a cut-off of 0.3 and 0.6 were higher in C1 than in C4, but the difference was not significant. Thus, CN C1 and C4 types were determined as the selection settings for future prediction models. The considerable difference in the average MCC values of C1 and C4 types and other consensus motifs was also potentially related to the overall CN ratio. Furthermore, we found that, as the negative ratio in types C1 and C4 increased, so did the average MCC values. In contrast, in types C2, C3, C5, and C6, an increase in the negative data ratio led to a decrease in MCC values. A potential reason for such findings is the relatively larger quantity of overall CN data in types C1 and C4. Although an increase in the negative data ratio reduced the prediction ability of positive data, machine learning maintained the prediction ability of positive data because of its sufficient quantity. Increased negative data increased the prediction ability of negative data. As a result, the MCC calculations showed an increase in overall prediction ability.

Figure 2b shows the MCC values in the CY model, in contrast to those for the CN model (Fig. 2a). The MCC was higher in types C1–C3 when compared with C4–C6, which suggests that an aspartic acid located two positions downstream from lysine in the consensus motif center can increase the MCC value by 0.04–0.06, thus substantially affecting consensus motif rules. Despite a mean MCC of 0.6 for type C2 (cut-off value of 0.3) in the CY model, the prediction performance of C2 in the CN model was poor (Fig. 2a). Therefore, C1 was selected as the consensus motif in this system.

### The selection of cut-off value and ratio in the C1 consensus motif

Once C1 was determined as the consensus motif, the cut-off value (0.3 or 0.6) and P/N ratio had to be selected. The settings with the highest MCC values in Table 3 were selected: a ratio of 1:2 in the CN model and 1:1 in the CY model. The cut-off value was equal to 0.3 in the CY model and all MCC values in the CN model were higher than the cut-off value of 0.6. For the final settings of the system, the cut-off value was thus set to 0.3 and the P/N ratios in the CN and CY models were set to 1:2 and 1:1, respectively.

**Table 3:** Average MCC values for the C1 motif under different cut-off values and P/N ratios.

Cut-off value	Consensus motif	Ratio (P/N)	Average MCC × 100
0.3	CN	1:1	40.482
		1:1.5	43.578
		1:2	**44.21**
	CY	1:1	**57.334**
		1:1.5	53.21
		1:2	46.8
0.6	CN	1:1	39.214
		1:1.5	41.983
		1:2	42.761
	CY	1:1	57.288
		1:1.5	54.696
		1:2	48.46

### Comparison of the results in the CNCY and noCNCY systems

**Figure 3:**
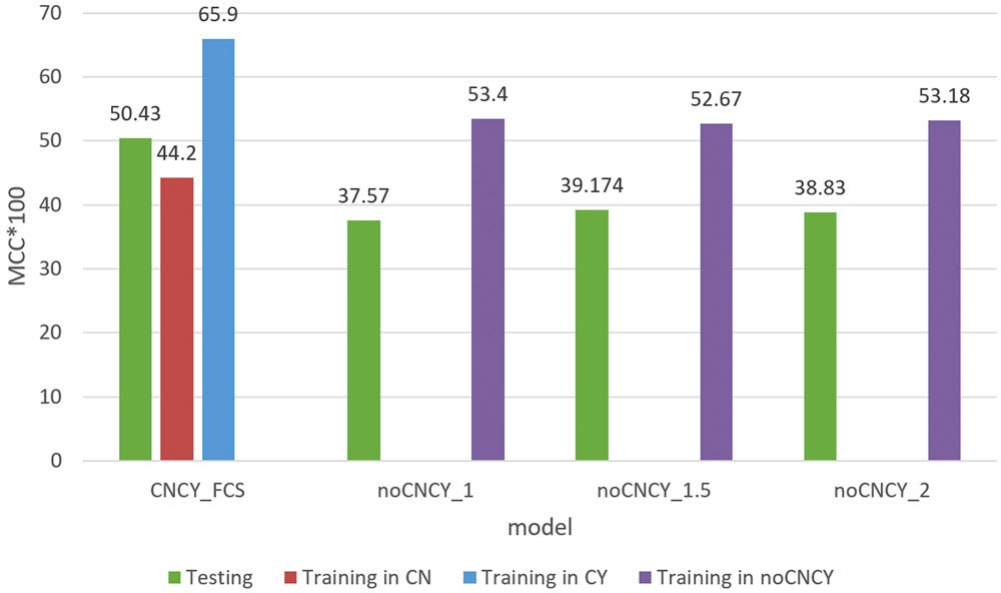
A comparison of MCC values resulting in the CNCY (with consensus motif classification) and noCNCY systems (without consensus motif classification).

To validate the benefits of the CNCY system in the construction of the prediction model, this study compared MCC values with and without consensus motifs; the results are presented in Fig. 3. With regard to the training data set, five-fold cross-validation showed that prediction results in cases when the CNCY system was not used (noCNCY) were higher than those of the CN model with the CNCY system. However, the MCC results of the testing data set were higher in the prediction model using the CNCY system than noCNCY system. These findings showed that despite the better prediction results of prediction models not using the CNCY system as based on cross-validation, the accuracy of such models was lower than that of models using the CNCY system when they were applied to data beyond the machine learning range.

### The results of excluding other PTM distribution features

To test the effect of the distribution of PTMs on the prediction model with respect to the prediction of SUMOylation, this study coded the model without PTM distribution features as “No mod” and applied it to the testing data set. The results are shown in Table 4. Table 4 is divided into three parts. The first part shows CNCY comparison results. The second part shows re-computation results obtained using the sum of TP, FP, TN, and FN in the CN and CY models. The third part shows the prediction results under a P/N ratio of 1:1. As seen from the first part, the accuracy of MCC values in prediction models was higher after adding PTM distribution features. The second part shows that the difference between the MCC values of the two models was 0.009 and that the MCC was higher in the system with PTM distribution features. As shown in the third part, with an equal ratio of positive and negative data, the system with PTM distribution features was more accurate. Thus, PTM distribution features can affect the accuracy of prediction systems.

**Table 4:** Testing data set results with and without PTM distribution features.

Item	Sn	Sp	ACC	Precision	MCC
No mod CN	0.375	0.983	0.963	0.433	0.384
No mod CY	0.573	0.917	0.781	0.818	0.535
FSC CN	0.39	0.983	0.964	0.438	0.395
FSC CY	0.579	0.918	0.785	0.821	**0.542**
**The results of combination with CN and CY**					
No mod	0.423	0.981	0.952	0.584	0.495
FSC	0.475	0.981	0.953	0.588	**0.504**
**Results from a P/N ratio of 1:1**					
No mod	0.846	0.685	0.741	0.589	0.505
FSC	0.85	0.687	0.744	0.592	**0.511**

### WEKA algorithm prediction model construction and comparison

With regard to the prediction results of the CN and CY models in the testing data set, the MCC of the SVM prediction model using the FSC mechanism reached 0.504, whereas the MCC of the CNCY prediction model constructed using the Random Forest algorithm in WEKA reached 0.52 (data not shown). Therefore, WEKA’s Random Forest was selected in this study as the prediction model algorithm and developed into the SUMOylation prediction tool SUMOgo.

### Comparison of other SUMOylation prediction tools

**Figure 4:**
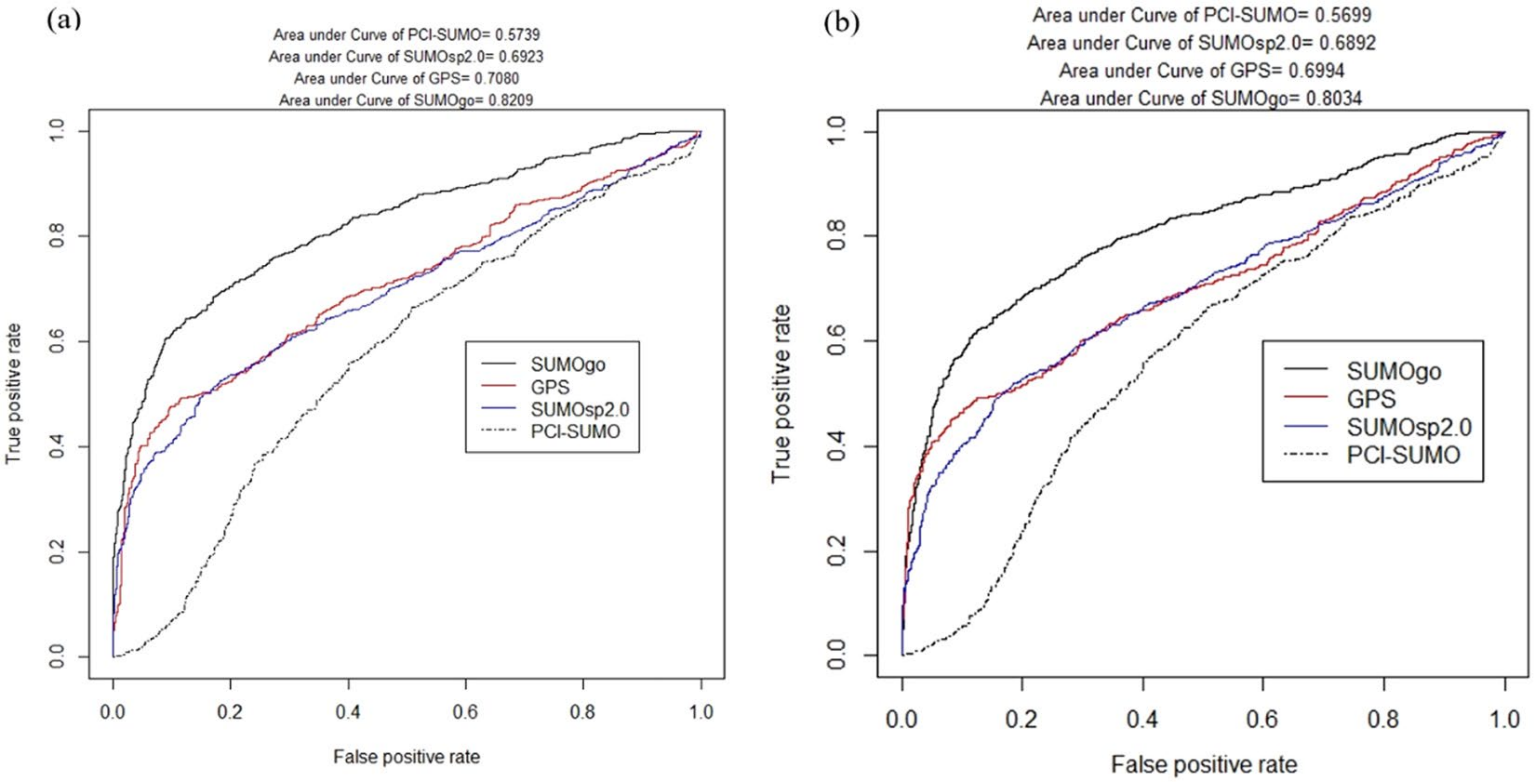
A ROC curve comparison of prediction results from SUMOgo with other SUMOylation prediction tools. (**a**) The best case and (**b**) worst case of area under curve of SUMOgo in the testing data set by conducting 20 cycles of random extraction of negative data at a 1:1 ratio.

This study compared the developed SUMOylation prediction tool SUMOgo and other prediction tools, including GPS-SUMO^1^, SUMOsp2.0^11^, JASSA^2^, and PCI-SUMO^15^. This study analyzed the testing data set with a P/N ratio of 1:1. The average MCC values and other evaluation indices of each prediction tool are presented in Table 5. The results indicated that SUMOgo showed the highest prediction accuracy and its average MCC value reached 0.511. As shown in Fig. 4, SUMOgo compared to the ROC curve of the other three tools, either best or worst performance, is superior to other SUMOylation prediction tools.

**Table 5:** Performance evaluation of each SUMOylation prediction tools.

	Sn	Sp	Acc	Precision	MCC
SUMOgo	0.592	0.896	0.744	0.850	**0.511**
GPS-L	0.668	0.810	0.739	0.778	0.482
GPS-M	0.642	0.833	0.738	0.794	0.484
GPS-H	0.540	0.897	0.719	0.840	0.468
SUMOsp2.0_L	0.709	0.750	0.730	0.739	0.460
SUMOsp2.0_M	0.655	0.823	0.739	0.787	0.485
SUMOsp2.0_H	0.608	0.873	0.740	0.827	0.498
JASSA	0.654	0.808	0.731	0.773	0.467
PCI-SUMO	0.687	0.530	0.609	0.594	0.220

### The ranking of feature selection

After feature selection, feature selection tools will rank the features in order of importance. Supplementary Table S1 shows the top ten features as determined by two feature selection tools within the CNCY system, LIBSVM and mRMR. The feature importance results from CNCY feature selection showed the high importance of the glutamic acid located in the 13th upstream position within the window size. The glutamic acid in this position is an important component of the consensus motif. Therefore, other features can be mined based on the position of this glutamic acid during the future development of prediction tools to enhance prediction accuracy.

In contrast to the LIBSVM feature selection, mRMR feature selection determined absolute surface accessibility as a more important feature for the CY, but not CN, model because of its potential effect on the ability of PTM-related proteins to attach to target proteins. Their inability to attach because of the position or area will result in the impossibility of PTM. With regard to feature selection in the CN model, absolute surface accessibility was also determined by mRMR as an important feature after the glutamic acid residue noted above. LIBSVM feature selection identified the 10th position in the window size as an important feature. As a result of the CN model rules, the 10th position in the window excludes four hydrophobic amino acids (L, V, M, and F). However, a comparison with hydrophobic amino acids selected in other studies showed the presence of five hydrophobic amino acids (A, I, P, G, and Y) in the CN model, which is the reason for the importance of the 10th position in the window. The remaining amino acid types may improve the CN model accuracy through one classification.

### Case study

CREB binding protein (CREBBP) is a multifunctional transcriptional coactivator. CREBBP was initially found to be a CREB coactivator. When the CREB transcription factor is phosphorylated by PKA, binding between CREBBP and CREB increases. As a result, CREBBP is bound by CREB to promoter regions and promotes CREB performance in gene activation^38,39^.

We used the SUMOgo prediction system to analyze this protein. Based on Q92793 (CBP_HUMAN) PTM features derived from UniProt, CREBBP has SUMOylation sites at amino acids 998, 1033, and 1056^40^. The SUMOgo prediction results identified 14 potential SUMOylation sites (Supplementary Table S2). Despite a larger quantity of false positive data, the three actual sites showed high reliability scores (0.860242, 0.820823, and 0.926328, respectively).

## Electronic supplementary material

Supplementary data

Dataset 1
