# Supplementary material for: SUMOgo: Prediction of sumoylation sites on lysines by motif screening models and the effects of various post-translational modifications
Source: Sci Rep. 2018 Oct 19;8:15512. doi: 10.1038/s41598-018-33951-5 (PMC6195521; doi:10.1038/s41598-018-33951-5)
Supplement: Supplementary file 1 — Supplementary data [file 41598_2018_33951_MOESM1_ESM.pdf]

SUMOgo: Prediction of sumoylation sites on lysines by motif screening models and the effects of various post-translational modifications

Chi-Chang Chang[1,2], Chi-Hua Tung[3], Chi-Wei Chen[4,5], Chin-Hau Tu[5] and Yen-Wei Chu[5,6,*]

[1]School of Medical Informatics, Chung-Shan Medical University, Taichung, Taiwan

[2]IT Office, Chung Shan Medical University Hospital, Taichung, Taiwan

[3]Department of Bioinformatics, Chung-Hua University, Rm. S116, 707, Sec.2, WuFu Rd., Hsinchu 30012, Taiwan

[4]Department of Computer Science and Engineering, National Chung-Hsing University 250, Kuo Kuang Rd., Taichung 402, Taiwan

[5]Institute of Genomics and Bioinformatics, National Chung Hsing University, 250, Kuo Kuang Rd., Taichung 402, Taiwan

[6]Biotechnology Center, Agricultural Biotechnology Center, Institute of Molecular Biology, National Chung Hsing University, 250, Kuo Kuang Rd., Taichung 402, Taiwan

*Corresponding. ywchu@nchu.edu.tw

Supplementary Materials

Table S1. The ranking of all feature selections

	LIBSVM feature selection		mRMR	
	CY	CN	CY	CN
1	257:Binary_p13_E_Glutamic acid	257:Binary_p13_E_Glutamic acid	717:Netsurf_p13_Absolute Surface Accessibility	257:Binary_p13_E_Glutamic acid
2	542:ph_p13_Secondary structure	193:Binary_p10_I_Isoleucine	257:Binary_p13_E_Glutamic acid	717:Netsurf_p13_Absolute Surface Accessibility
3	548:ph_p13_apha_helix propensity	516:ph_p10_Hydrophobicity	752:Netsurf_p18_Absolute Surface Accessibility	193:Binary_p10_I_Isoleucine
4	550:ph_p13_beta_strand propensity	550:ph_p13_beta_strand propensity	689:Netsurf_p9_Absolute Surface Accessibility	682:Netsurf_p8_Absolute Surface Accessibility
5	547:ph_p13_Side chain length	872:ModPred_SUMOylation_p11	633:Netsurf_p1_Absolute Surface Accessibility	745:Netsurf_p17_Absolute Surface Accessibility
6	541:ph_p13_Polarity	511:ph_p10_Polarity	542:ph_p13_Secondary structure	766:Netsurf_p20_Absolute Surface Accessibility
7	549:ph_p13_Number of codons	548:ph_p13_apha_helix propensity	724:Netsurf_p14_Absolute Surface Accessibility	696:Netsurf_p10_Absolute Surface Accessibility
8	543:ph_p13_Molecular size or volume	549:ph_p13_Number of codons	668:Netsurf_p6_Absolute Surface Accessibility	773:Netsurf_p21_Absolute Surface Accessibility
9	717:Netsurf_p13_Absolute Surface Accessibility	542:ph_p13_Secondary structure	731:Netsurf_p15_Absolute Surface Accessibility	710:Netsurf_p12_Absolute Surface Accessibility
10	540:ph_p12_beta_strand propensity	541:ph_p13_Polarity	738:Netsurf_p16_Absolute Surface Accessibility	513:ph_p10_Molecular size or volume

Table S2. SUMOgo prediction results on CREB binding protein

**Position**	**Confidence Score**
1056	0.926328
1060	0.875346
998	0.860242
1033	0.820823
1042	0.792598
1086	0.740013
1052	0.732580
1014	0.692519
315	0.647038
1203	0.646879
1565	0.644740
2102	0.633585
635	0.624053
1564	0.591478
